# Early intervention, lasting impact: benefits of early antiretroviral therapy and implications for posttreatment control

**DOI:** 10.1097/COH.0000000000001040

**Published:** 2026-05-15

**Authors:** Dita C. Bolluyt, Godelieve J. de Bree, Alexander O. Pasternak

**Affiliations:** a Laboratory for Experimental Virology, Department of Medical Microbiology and Infection Prevention; b Amsterdam Institute for Infection and Immunity; c Department of Internal Medicine, section Infectious Diseases, Amsterdam UMC, Amsterdam, The Netherlands

**Keywords:** acute HIV infection, early antiretroviral therapy, HIV cure, HIV reservoir, HIV-specific immune response

## Abstract

**Purpose of review:**

Over the past few years, it has become increasingly clear that starting antiretroviral therapy (ART) during acute HIV infection (AHI) leads to a faster viral reservoir decay and partial preservation of the immune system. Here, we review the benefits of treating AHI for the reservoir composition and the adaptive immune function, as well as implications for HIV cure studies.

**Recent findings:**

AHI is most commonly defined as the first 6 months following infection. During this early infection stage of infection, HIV viremia reaches its peak, concurrently with the development of the HIV-specific immune response. Early ART limits the size of the viral reservoir, and a smaller reservoir is associated with of longer time to viral rebound posttreatment interruption and, in some cases, posttreatment control (PTC). Moreover, initiating ART during AHI partially preserves the function of T and B cells, leading to improved control of the viral reservoir, and potentially creating windows for HIV cure strategies.

**Summary:**

Recent cure studies show that both CD8^+^ T-cell and antibody responses provide important clues for predicting PTC, as individuals with robust, functional immune profiles are more likely to maintain viral suppression after stopping ART. However, while early ART initiation lowers the viral reservoir and preserves immune function, these factors alone are not sufficient for PTC, highlighting the need for a comprehensive molecular profile that integrates viral and host biomarkers to reliably predict PTC.

## INTRODUCTION

Combination antiretroviral therapy (ART) can successfully suppress HIV replication, partially restore immune function, and prevent the development of AIDS [1]. However, ART cannot eliminate HIV infection and therefore treatment interruption results in rapid viral rebound, originating from latent HIV reservoirs. Consequently, people with HIV (PWH) must commit to lifelong therapies, facing chronic immune activation and inflammation as well as drug toxicities. Therefore, there is an urgent need to develop safe, affordable, and globally accessible curative strategies. Although a definitive cure is not yet available, growing evidence suggests that PWH who initiate ART during the acute phase of HIV infection (AHI) may be particularly well positioned to benefit from experimental cure strategies. AHI is defined as the first 6 months of HIV infection and is subclassified into Fiebig stages [2]. During AHI, plasma viremia reaches a peak, and although the emerging HIV-specific immune response is insufficient to fully control viral replication, it starts to exert pressure on the virus. This interaction ultimately results in stabilization of viral load at a relatively steady-state level, referred to as the viral set point [3].

Several clinical studies have demonstrated that intermittent ART during AHI is associated with improved immune recovery, including higher likelihood of achieving and maintaining higher CD4^+^ T-cell counts, lower viral set point, and reduced viral reservoir size compared to deferred therapy [4–7]. In these studies, intermittent ART demonstrated transient benefits after analytical treatment interruption (ATI), including delayed CD4+ T-cell decline and postponement of ART reinitiation, although these effects waned over time. These observations led to adjustment of treatment guidelines to start ART in the early phase of infection [8].

In recent years, it became apparent that, beyond its clinical benefit, ART initiated during AHI can also enhance the effectiveness of experimental curative interventions. With the establishment of cohorts of PWH who initiated ART during AHI, it became evident that early treatment shapes both the size and characteristics of the viral reservoir, as well as the maturation and quality of the HIV-specific immune response [4–7,9–13,14,15,16,17^▪^]. Furthermore, PWH who start treatment during AHI are more likely to control HIV upon ATI after temporary ART, also termed posttreatment control (PTC) [18^▪▪^]. ATIs are now essential components of cure trials, as they enable to assess postintervention control (PIC) using endpoints such as time to viral rebound and control of viral load [19–21]. As of today, these trials have shown limited success, with most participants facing viral rebound. Therefore, a better understanding of the viro-immunological factors modulated by early ART initiation that contribute to PTC is essential [22,23].

Here, we review the benefits of AHI treatment for reservoir composition and the adaptive immune function, as well as implications for PTC in HIV cure studies. 

**Box 1:**
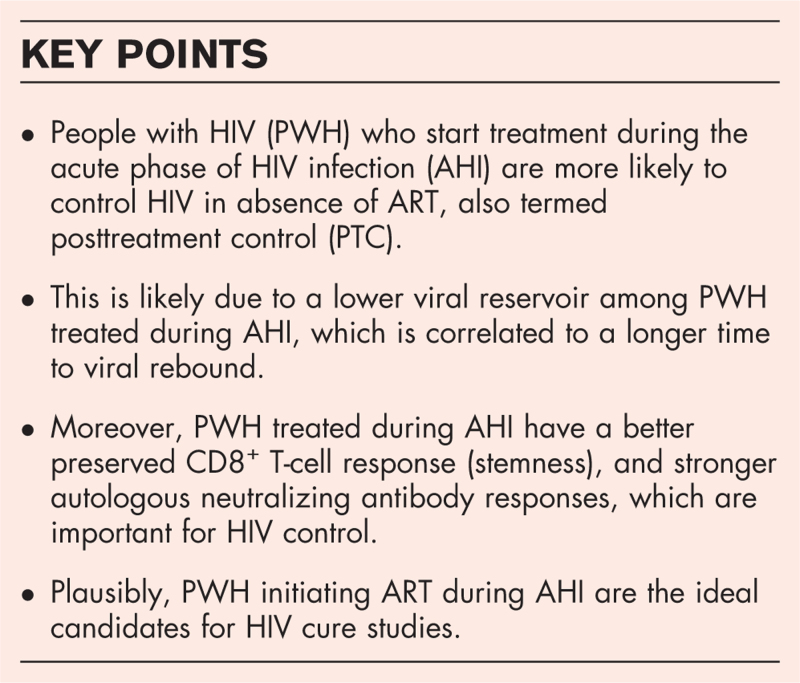
no caption available

## BENEFITS OF EARLY ANTIRETROVIRAL THERAPY FOR REDUCING THE VIRAL RESERVOIR

ART is not curative due to the persistence of long-lived viral reservoirs [24,25]. The main reservoir is thought to reside in latently infected resting CD4^+^ T cells in peripheral blood and lymphoid tissue, although other cell types such as macrophages may contribute as well [26,27]. Even after many years of successful treatment, ART interruption typically leads to a viral rebound within 2–4 weeks [28]. Regardless of initiating ART within the first days after HIV infection, the viral reservoir will be established, and rebound will occur in most PWH upon stopping treatment.

Due to integration into the host genome, the provirus will persist under ART in these reservoirs as a result of continuous proliferation of the infected cells [29]. Most of the integrated HIV DNA is replication-defective [30]. After prolonged ART, intact proviruses are frequently found in transcriptionally silent regions of the host genome (heterochromatin regions), hidden from the immune system [31]. It has recently been shown that even before peak viremia, there are translationally inactive proviruses, suggesting that deep latency can be established during AHI [32]. Indeed, a recent report showed transcriptional blocks were already present in untreated early infection [33^▪^]. Another study detected provirus in the heterochromatin regions among AHI treated after 1 year, which are enriched over time, suggesting clonal expansion of cells with proviruses integrated in this region [34^▪^]. Therefore, early ART initiation is not sufficient for a cure.

However, PTC is observed more frequently in early treated PWH compared to those initiating ART during chronic HIV infection (CHI) [18^▪▪^,^35^], suggesting that early intervention may increase the likelihood of PTC, even though it does not guarantee a cure. Multiple studies have shown that early ART leads to a steeper decline and lower reservoirs compared to CHI ART [16,36–45]. Low reservoirs have been correlated to a longer time to viral rebound and even PTC [15,46–49], indicating an important beneficial effect of early treatment. A mathematical modeling study using long-term data of PWH treated during AHI also observed a steep initial reservoir decay when initiating ART during AHI compared to CHI, which is even faster when initiating ART within the first 11 weeks after infection [50^▪^]. Moreover, this study observed a significantly faster decay of defective compared to intact HIV DNA during the initial 5 weeks of ART. Our group recently demonstrated a significant decline in defective but not intact HIV DNA between 24 and 156 weeks after early ART initiation [51^▪^], something that is usually not observed in PWH treated during CHI [52,53]. Defective proviruses can be still transcription-competent and translation-competent, playing a role in ongoing immune activation under ART [54–59]. Interestingly, Reddy *et al.*[60^▪^] found a faster decay of intact compared to defective genomes during the first year on ART, which was more profound among PWH treated at AHI. In this study, intact genomes persisted after 1 year of CHI ART but were undetectable after 1 year of early ART.

Another important benefit of early treatment is its long-term effect on the viral reservoir. In the Primo-SHM cohort of PWH who have been treated with 24 or 60 weeks of ART during primary HIV infection [4], upon interruption of early treatment and subsequent ART reinitiation after a median of 116  weeks without ART, HIV reservoir markers returned to their preinterruption levels on early treatment [61^▪▪^]. Moreover, levels of HIV reservoir markers during CHI ART were lower in participants who had been temporarily pretreated during AHI, compared to those who have been randomized not to receive early ART [61^▪▪^]. This implies a long-term effect of temporary early ART on the reservoir, revealed during therapy reinitiated after several years. In a related vein, a large multinational trial with 20 years of follow-up showed a lower hazard of viral ‘blips’ when ART was initiated in the first 2 years of infection, indicating a long-term benefit of early ART for the viral reservoir [62^▪^].

Overall, early ART leads to lower reservoirs, which may increase the likelihood of achieving PTC. However, even in PWH treated during the hyperacute phase (Fiebig I-II, before peak viremia), PTC is not achieved in the majority of cases, and heterogeneity in reservoir size is observed [28,63]. This suggests other factors than mere timing of ART initiation play a role in the development of PTC.

## BENEFITS OF EARLY ANTIRETROVIRAL THERAPY FOR PRESERVING THE ADAPTIVE IMMUNE FUNCTION

### CD8^+^ T cells

In the early stage of untreated HIV infection, an HIV-specific CD8^+^ T-cell response emerges. It has been shown that a stronger proliferative capacity of HIV-specific CD8^+^ T cells during early infection is associated with a lower viral set point [13]. Moreover, during AHI, HIV-specific CD8^+^ T cells are more cytotoxic compared to CHI [64]. When ART is initiated during AHI, CD8^+^ T cells remain relatively functional, can reduce reservoir seeding, and less HIV-specific CD8^+^ T-cell escape occurs [60^▪^,^65^]. In the absence of ART, however, persistent antigen exposure drives continued T-cell proliferation, leading to CD8^+^ T-cell exhaustion [66]. A key question addressed in the past years has been to what extent early ART, by limiting antigen exposure, shapes the magnitude, functionality, and differentiation profile of the HIV-specific CD8^+^ T-cell response. Several groups have addressed that subject in diverse acute treated cohorts.

Initiation of ART in hyperacute infection preserved CD4^+^ T-cell help and maintained functional HIV-specific CD8^+^ T-cell responses [65,67]. In individuals who initiated ART in the hyperacute infection phase, HIV-specific CD8^+^ T cells exhibited increased CD127 (pro-survival) and BCL-2 (antiapoptotic) expression, enhanced in-vitro IFN-γ production, and increased differentiation into an effector memory phenotype, which is more effective in controlling viral infection [67]. Transcriptional profiling of these HIV-specific CD8^+^ T cells further demonstrated, compared to individuals that start ART during CHI, reduced expression of genes linked to activation and apoptosis, alongside upregulation of pro-survival pathways, including BCL-2. Takata *et al.* [68] also found increased CD127 expression among AHI, together with increased TCF-1 expression, which is associated with a phenotype of long-lived memory T cells with capacity to self-renewal (referred to as stemness). In CHI, HIV-specific CD8^+^ T cells exhibited a phenotype skewed toward short-lived memory cells, accompanied by increased expression of PD-1, a marker of T-cell exhaustion. In addition, when examining CD8^+^ T cells that target the lymph node-localized viral reservoir – follicular (CXCR5^+^) CD8^+^ T cells [69] – ART initiated during AHI was associated with a superior antiviral function compared to ART started during CHI, and an inverse correlation between follicular CD8^+^ T cells and the size of the intact proviral reservoir was measured [70^▪^]. Importantly, CD8^+^ T-cell characteristics depend on the Fiebig stage at ART initiation. When ART is initiated before peak viremia, HIV-specific CD8^+^ T cells expand more slowly and acquire effector functions later but display an enhanced memory potential. In contrast, HIV-specific CD8^+^ T cells primed at peak viremia (Fiebig III) are more terminally differentiated and apoptosis-prone [65].

Growing evidence indicates that CD8^+^ T cells recognize viral reservoir, even defective reservoir [71] and constrain both the size and transcriptional activity of the viral reservoir, particularly in individuals who initiate ART at AHI. Combined data from the RV254/SEARCH010 and the RV304/SEARCH013 cohorts showed that a strong and sustained HIV-specific CD8^+^ T-cell response was linked to a greater decline in integrated HIV DNA [72]. However, larger numbers of HIV-specific CD8^+^ T cells are also associated with a larger active viral reservoir during ART, as reflected by cell-associated RNA. This study suggests that the active viral reservoir helps maintain the number of HIV-specific CD8^+^ T cells, but at the same time limits their maturation into fully functional, effective cells [72]. In the Netherlands Cohort Study on Acute HIV infection (NOVA) [9], we demonstrated that the decline in total and defective HIV DNA between 24 and 156 weeks of immediate ART was predicted by the HIV-specific CD8^+^ T-cell proliferative response at 24 weeks after early ART initiation [51^▪^]. A separate study in the Primo-SHM cohort found an inverse correlation of CD8^+^ T-cell responses with intact HIV DNA, suggesting that the CD8^+^ T cells restrict the intact reservoir [61^▪▪^]. A similar negative correlation between HIV-specific (Gag) CD8^+^ T cells and intact viral reservoir was observed by Li *et al. *[73^▪^]. Moreover, Rueger *et al.*[70^▪^] found that early treatment preserved effector functions of these CD8^+^ T cells, which was correlated to reservoir decline.

Altogether, these data suggest that HIV-specific CD8^+^ T-cell response is maintained by the viral reservoir. Together with this, under ART initiated during AHI, these CD8^+^ T cells retain functionality with stemlike properties and can exert immune pressure that contributes to the decay of the viral reservoir.

### B cells and antibodies

Neutralizing antibodies (nAbs) are a central component of antiviral adaptive immunity [74] and contribute significantly to the immune response against HIV. The antibody response directed against HIV can be categorized into autologous NAbs (aNAbs) and broadly NAbs (bNAbs). aNAbs emerge early after infection and primarily recognize the individual's circulating viral strain [75]. However, their effectiveness is limited by rapid viral escape. In contrast, bNAbs typically develop years after infection, target conserved epitopes on the viral envelope, exhibit cross-strain neutralizing activity, and have demonstrated the capacity to suppress viremia (reviewed in [76]). From the perspective of the formation of adaptive immunity in AHI, and implications for cure interventions, we will focus on the development of aNAbs.

aNAbs primarily target the HIV envelope glycoprotein (Env) expressed on virions and infected cells. They arise during AHI and increase in potency over subsequent months [77]. However, viral escape through Env mutation, which already occurs during AHI, limits the capacity of aNAbs for durable viral control [75,77]. There is evidence that indicates aNAbs can maintain immune pressure and that these responses continue to mature under ART initiated during AHI. In individuals treated during AHI, ART initiation permitted maturation of aNAb responses that exert selective pressure on rebounding virus following treatment interruption [78].

B cells and plasma cells are the producers of aNAbs. From the earliest stages of infection, memory B cells become exhausted and shift towards immature B cells. Moreover, due to CD4^+^ T-cell depletion in CHI, B cells lack CD4^+^ T-cell help, which drives them into apoptosis [79]. Initiation of ART during AHI results in better preservation of B-cell function [80]. An important remaining question is whether this improved preservation also translates into a more robust and effective aNAb response. A recent study showed that aNAbs against Env are stronger when ART started 60 days after infection compared to starting in the acute phase (<60 days) [81^▪^]. These data suggest that a certain level of viral exposure may be required for the development of effective anti-HIV antibody responses. This concept is supported by observations in individuals treated during AHI who showed delayed formation of nAbs, which has been linked to limited germinal center development at the time of early ART initiation [82^▪▪^]. In lymph node biopsy samples, activation of CXCR3^+^ T-follicular helper cells – an essential step in early germinal center formation – was observed to begin only after peak viremia. This provides a plausible explanation why PWH initiating ART in the hyperacute phase may not produce adequate aNAbs. At the same time, early treatment limits the establishment of viral immune escape variants and therefore maintains low Env diversity, which increases the likelihood that the virus remains sensitive to bNAbs – antibodies that are being investigated as interventions in HIV cure trials – compared to individuals who initiate treatment during CHI [83^▪^].

## IMPACT OF THE ADAPTIVE IMMUNE RESPONSE ON DETERMINANTS OF POST-TREATMENT CONTROL IN CURE STUDIES

One of the scenarios for an HIV cure is control of the virus in absence of ART (termed HIV remission or functional cure), which seems more feasible than the complete eradication of HIV [25]. PTCs can control HIV replication after therapy interruption for extended periods and thus may represent a model of long-term HIV remission without ART. Three recent cure trials that included PWH treated at AHI, identified a similar pattern following ATI: some participants experienced a rapid viral rebound, others exhibited a delayed rebound with oscillating viral load, and a subset maintained sustained viral suppression throughout the follow-up period [84,85,86^▪▪^]. While the underlying mechanisms remain under investigation, the recurrence of these patterns in three independent trials suggests an interplay between the host immune system and HIV, wherein a certain immune profile might be beneficial.

One of the reasons why PTC is more frequent after early ART seems to be the lower viral reservoir among PWH treated during AHI [73^▪^]. However, a low reservoir seems to be necessary but not sufficient for PTC, considering the majority of early treated PWH, or those with a low reservoir, still do not achieve PTC [18^▪▪^]. A study comparing PTCs (both AHI and CHI) to noncontrollers (NCs) found significant lower CD4^+^ and CD8^+^ T-cell activation and CD4^+^ T-cell exhaustion after ATI in PTCs, highlighting the importance of T-cell functionality in PTC [87]. A recent study that investigated PICs after bNAb intervention trials showed an enrichment of HIV-specific CD8^+^ T cells with a ‘stem-cell like state', which was boosted by the bNAbs, in PICs [88^▪▪^]. A similar CD8^+^ T-cell profile was described in an intervention study following vaccination to boost T-cell responses in combination with two bNAbs and a latency reversal agent. In this study, PIC was mostly attributed to an expansion of these stemlike T cells [86^▪▪^]. Collectively, these studies point in the direction of a role for CD8^+^ T-cell function in predicting successful PIC.

Accumulating evidence indicates that aNAbs contribute to shaping viral rebound and may distinguish PTCs from NCs. In individuals treated during AHI, aNAb responses around time of ATI exerted measurable selective pressure, as rebounding viruses were more resistant to post-ATI plasma neutralization than pre-ART variants [78]. Moreover, several pre-ATI features differentiated PTCs from NCs, including more restricted proviral diversity, and stronger aNAb responses. Rebound viruses have been shown to originate predominantly from intact proviruses within resting CD4^+^ T cells and over time, rebounding viruses were resistant to IgG-mediated neutralization. These data suggest that to some extent, the viral reservoir is kept under control by aNAbs [89^▪^]. However, not all studies support a clear predictive role for humoral immunity as determinant for rebound. In a study in CHI, no differences in Env-binding titers, Fc receptor binding, or antibody effector functions (ADCP, ADNP, ADNKA) were observed between PTCs and NCs [90^▪^]. Similarly, enhanced functional humoral immunity at ATI correlated with delayed rebound in a chronically treated cohort, but this association was not observed in an acute-treated cohort and likely related to viral activation in the chronic cohort [91]. Finally, coordinated antibody titers and functional activity have been described in established PTCs [92], although these measurements were obtained during the control phase rather than prior to ATI. Collectively, these findings suggest that aNAb quantity and quality may influence rebound dynamics, but their predictive value depends on timing of ART initiation and reservoir characteristics.

## CONCLUSION

**FIGURE 1:**
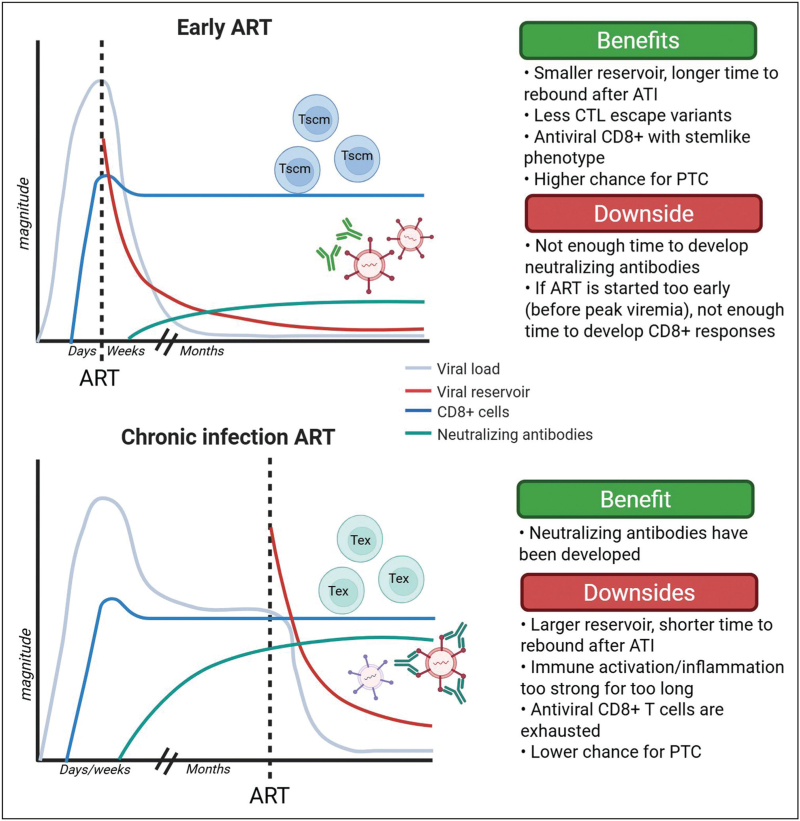
Benefits and downsides of initiating antiretroviral therapy during the acute or chronic phase of HIV infection. The approximate dynamics of plasma viral load, viral reservoir, CD8^+^ T cells, and neutralizing antibodies are shown with lines. Depicted are the CD8^+^ T-cell phenotypes as well as neutralizing antibodies, which are mostly developed during the chronic infection, however, viral escape variants are observed. ATI, analytical treatment interruption; CTL, cytotoxic T lymphocytes; PTC, posttreatment control; Tex, CD8^+^ T cells with an exhausted phenotype; Tscm, CD8^+^ T cells with a stem-cell like phenotype. Created with Biorender.

PWH initiating ART during AHI harbor lower reservoirs and better preserved T-cell and B-cell functionality, making them ideal candidates for cure studies (Fig. 1). Moreover, these benefits make them more likely to control HIV in absence of ART. However, as early ART initiation is not sufficient for PTC or PIC, further research is needed to define a molecular profile predictive of HIV remission [93]. Such a profile, incorporating multiple viral and host biomarkers, should be able to reliably predict PTC and ideally offer a personalized treatment and follow-up plan. Parallel assessment of proviral genetic intactness, chromatin context of the integration site, and HIV transcriptional activity at the single-proviral level, together with the assessment of innate and adaptive host immune pressures on the reservoir could provide a predictive score for PTC. Such a composite molecular profile is expected to outperform each of the individual component biomarkers by the predictive power for viral rebound. Learning more about the difference between PWH who experience fast viral rebound after treatment interruption and those who demonstrate some degree of PTC may also reveal relevant biomarkers for PIC. Identifying the genetic, immunological, and virological predictors and correlates of PTC will advance our knowledge of viral control and is critical for designing cure-related clinical trials.

## Acknowledgements

*None*.

### Financial support and sponsorship

*A.O.P. acknowledges grant support from amfAR, The Foundation for AIDS Research (grant no. 1110680-77-RPRL), and from Partnership NWO-Dutch AIDS Fonds ‘HIV cure for everyone’ (grant no. KICH2.V4P.AF23.001).*

### Conflicts of interest

*There are no conflicts of interest.*

## REFERENCES AND RECOMMENDED READING

Papers of particular interest, published within the annual period of review, have been highlighted as:

▪ of special interest
▪▪ of outstanding interest

## References

[1] Deeks SG Lewin SR Havlir DV. The end of AIDS: HIV infection as a chronic disease. Lancet 2013; 382:1525–1533. 24152939 10.1016/S0140-6736(13)61809-7 PMC4058441

[2] Fiebig EW Wright DJ Rawal BD. Dynamics of HIV viremia and antibody seroconversion in plasma donors: implications for diagnosis and staging of primary HIV infection. AIDS 2003; 17:1871–1879. 12960819 10.1097/00002030-200309050-00005

[3] Deeks SG Overbaugh J Phillips A Buchbinder S. HIV infection. Nat Rev Dis Primers 2015; 1:15035. 27188527 10.1038/nrdp.2015.35

[4] Grijsen ML Steingrover R Wit FW. No treatment versus 24 or 60 weeks of antiretroviral treatment during primary HIV infection: the randomized Primo-SHM trial. PLoS Med 2012; 9:e1001196. 22479156 10.1371/journal.pmed.1001196 PMC3313945

[5] Fidler S Porter K Spartac Trial Investigators. Short-course antiretroviral therapy in primary HIV infection. N Engl J Med 2013; 368:207–217. 23323897 10.1056/NEJMoa1110039 PMC4131004

[6] Hecht FM Wang L Collier A AIEDRP Network. A multicenter observational study of the potential benefits of initiating combination antiretroviral therapy during acute HIV infection. J Infect Dis 2006; 194:725–733. 16941337 10.1086/506616

[7] Hogan CM Degruttola V Sun X A5217 Study Team. The setpoint study (ACTG A5217): effect of immediate versus deferred antiretroviral therapy on virologic set point in recently HIV-1-infected individuals. J Infect Dis 2012; 205:87–96. 22180621 10.1093/infdis/jir699 PMC3242744

[8] World Health Organization. Guideline on When to Start Antiretroviral Therapy and on Pre-Exposure Prophylaxis for HIV. WHO Guidelines Approved by the Guidelines Review Committee. Geneva, 2015. 26598776

[9] Dijkstra M Prins H Prins JM. Cohort profile: the Netherlands Cohort Study on Acute HIV infection (NOVA), a prospective cohort study of people with acute or early HIV infection who immediately initiate HIV treatment. BMJ Open 2021; 11:e048582. 10.1136/bmjopen-2020-048582 PMC8634014 34845066

[10] De Clercq J De Scheerder MA Vanherrewege S. Staging of immuno-virological dynamics during acute HIV infection in a Belgian prospective cohort study. J Virus Erad 2024; 10:100392. 39403428 10.1016/j.jve.2024.100392 PMC11472225

[11] Freind MC Tallon de Lara C Kouyos RD. Cohort profile: the Zurich Primary HIV Infection Study. Microorganisms 2024; 12:302. 38399706 10.3390/microorganisms12020302 PMC10893142

[12] Robb ML Eller LA Kibuuka H RV 217 Study Team. Prospective study of acute HIV-1 infection in adults in East Africa and Thailand. N Engl J Med 2016; 374:2120–2130. 27192360 10.1056/NEJMoa1508952 PMC5111628

[13] Ndhlovu ZM Kamya P Mewalal N. Magnitude and kinetics of CD8+ T-cell activation during hyperacute HIV infection impact viral set point. Immunity 2015; 43:591–604. 26362266 10.1016/j.immuni.2015.08.012 PMC4575777

[14▪] Crowell TA Ritz J Zheng L AIDS Clinical Trials Group (ACTG) A5354/EARLIER Study Team. Impact of antiretroviral therapy during acute or early HIV infection on virologic and immunologic outcomes: results from a multinational clinical trial. AIDS 2024; 38:1141–1152. 38489580 10.1097/QAD.0000000000003881 PMC11323228

[15] Saez-Cirion A Bacchus C Hocqueloux L. Posttreatment HIV-1 controllers with a long-term virological remission after the interruption of early initiated antiretroviral therapy ANRS VISCONTI Study. PLoS Pathog 2013; 9:e1003211. 23516360 10.1371/journal.ppat.1003211 PMC3597518

[16] Massanella M Bender Ignacio RA Lama JR. Long-term effects of early antiretroviral initiation on HIV reservoir markers: a longitudinal analysis of the MERLIN clinical study. Lancet Microbe 2021; 2:e198–e209. 10.1016/S2666-5247(21)00010-0 35544209

[17▪] Moura SS Caetano DG Guimaraes ML. Acute HIV infection and ART response: insights into T cell subsets, activation, exhaustion, and blood and GALT HIV reservoir. Viruses 2025; 17:1381. 41157651 10.3390/v17101381 PMC12568170

[18▪▪] Gunst JD Gohil J Li JZ. Time to HIV viral rebound and frequency of posttreatment control after analytical interruption of antiretroviral therapy: an individual data-based meta-analysis of 24 prospective studies. Nat Commun 2025; 16:906. 39837813 10.1038/s41467-025-56116-1 PMC11751076

[19] Julg B Dee L Ananworanich J. Recommendations for analytical antiretroviral treatment interruptions in HIV research trials-report of a consensus meeting. Lancet HIV 2019; 6:e259–e268. 30885693 10.1016/S2352-3018(19)30052-9 PMC6688772

[20] Sandel DA Rutishauser RL Peluso MJ. Postintervention control in HIV immunotherapy trials. Curr Opin HIV AIDS 2025; 20:70–79. 39494630 10.1097/COH.0000000000000890 PMC11620322

[21] Pitman MC Lau JSY McMahon JH Lewin SR. Barriers and strategies to achieve a cure for HIV. Lancet HIV 2018; 5:e317–e328. 29893245 10.1016/S2352-3018(18)30039-0 PMC6559798

[22] Giron LB Pasternak AO Abdel-Mohsen M. Soluble markers of viral rebound and posttreatment HIV control. Curr Opin HIV AIDS 2025; 20:61–69. 39392413 10.1097/COH.0000000000000889 PMC11620946

[23] Mesquita FS Li Y Li JZ. Viral and immune predictors of HIV posttreatment control. Curr Opin HIV AIDS 2025; 20:54–60. 39633539 10.1097/COH.0000000000000898 PMC13157257

[24] Deeks SG Archin N Cannon P. Research priorities for an HIV cure: International AIDS Society Global Scientific Strategy. Nat Med 2021; 27:2085–2098. 34848888 10.1038/s41591-021-01590-5

[25] Ndung’u T McCune JM Deeks SG. Why and where an HIV cure is needed and how it might be achieved. Nature 2019; 576:397–405. 31853080 10.1038/s41586-019-1841-8 PMC8052635

[26] Fromentin R Chomont N. HIV persistence in subsets of CD4+ T cells: 50 shades of reservoirs. Semin Immunol 2021; 51:101438. 33272901 10.1016/j.smim.2020.101438 PMC8164644

[27] Chen W Berkhout B Pasternak AO. Phenotyping viral reservoirs to reveal HIV-1 hiding places. Curr HIV/AIDS Rep 2025; 22:15. 39903363 10.1007/s11904-025-00723-6 PMC11794352

[28] Colby DJ Trautmann L Pinyakorn S RV411 study group. Rapid HIV RNA rebound after antiretroviral treatment interruption in persons durably suppressed in Fiebig I acute HIV infection. Nat Med 2018; 24:923–926. 29892063 10.1038/s41591-018-0026-6 PMC6092240

[29] McMyn NF Varriale J Fray EJ. The latent reservoir of inducible, infectious HIV-1 does not decrease despite decades of antiretroviral therapy. J Clin Invest 2023; 133:e171554. 37463049 10.1172/JCI171554 PMC10471168

[30] Bruner KM Murray AJ Pollack RA. Defective proviruses rapidly accumulate during acute HIV-1 infection. Nat Med 2016; 22:1043–1049. 27500724 10.1038/nm.4156 PMC5014606

[31] Lian X Seiger KW Parsons EM. Progressive transformation of the HIV-1 reservoir cell profile over two decades of antiviral therapy. Cell Host Microbe 2023; 31:83.e5–96.e5. 36596305 10.1016/j.chom.2022.12.002 PMC9839361

[32] Gantner P Buranapraditkun S Pagliuzza A. HIV rapidly targets a diverse pool of CD4(+) T cells to establish productive and latent infections. Immunity 2023; 56:653.e5–668.e5. 36804957 10.1016/j.immuni.2023.01.030 PMC10023508

[33▪] Janssens J Wedrychowski A Kim SJ. Longitudinal changes in the transcriptionally active and intact HIV reservoir after starting ART during acute infection. J Virol 2025; 99:e0143124. 39907283 10.1128/jvi.01431-24 PMC11915860

[34▪] Struyve T Pardons M De Clercq J. Evolution of the HIV-1 integration site landscape and inducible reservoir in early-treated people. PLoS Pathog 2025; 21:e1013702. 41289275 10.1371/journal.ppat.1013702 PMC12646413

[35] Namazi G Fajnzylber JM Aga E. The Control of HIV After Antiretroviral Medication Pause (CHAMP) Study: posttreatment controllers identified from 14 clinical studies. J Infect Dis 2018; 218:1954–1963. 30085241 10.1093/infdis/jiy479 PMC6217727

[36] Leyre L Kroon E Vandergeeten C. Abundant HIV-infected cells in blood and tissues are rapidly cleared upon ART initiation during acute HIV infection. Sci Transl Med 2020; 12:eaav3491. 32132218 10.1126/scitranslmed.aav3491 PMC7293182

[37] van Zyl GU Bedison MA van Rensburg AJ. Early antiretroviral therapy in South African children reduces HIV-1-infected cells and cell-associated HIV-1 RNA in blood mononuclear cells. J Infect Dis 2015; 212:39–43. 25538273 10.1093/infdis/jiu827 PMC4542592

[38] Malatinkova E De Spiegelaere W Bonczkowski P. Impact of a decade of successful antiretroviral therapy initiated at HIV-1 seroconversion on blood and rectal reservoirs. Elife 2015; 4:e09115. 26439007 10.7554/eLife.09115 PMC4657623

[39] Schmid A Gianella S von Wyl V. Profound depletion of HIV-1 transcription in patients initiating antiretroviral therapy during acute infection. PLoS One 2010; 5:e13310. 20967271 10.1371/journal.pone.0013310 PMC2953504

[40] Buzon MJ Martin-Gayo E Pereyra F. Long-term antiretroviral treatment initiated at primary HIV-1 infection affects the size, composition, and decay kinetics of the reservoir of HIV-1-infected CD4 T cells. J Virol 2014; 88:10056–10065. 24965451 10.1128/JVI.01046-14 PMC4136362

[41] Jain V Hartogensis W Bacchetti P. Antiretroviral therapy initiated within 6 months of HIV infection is associated with lower T-cell activation and smaller HIV reservoir size. J Infect Dis 2013; 208:1202–1211. 23852127 10.1093/infdis/jit311 PMC3778965

[42] Strain MC Little SJ Daar ES. Effect of treatment, during primary infection, on establishment and clearance of cellular reservoirs of HIV-1. J Infect Dis 2005; 191:1410–1418. 15809898 10.1086/428777

[43] Yerly S Kaiser L Perneger TV. Time of initiation of antiretroviral therapy: impact on HIV-1 viraemia. The Swiss HIV Cohort Study. AIDS 2000; 14:243–249. 10716500 10.1097/00002030-200002180-00006

[44] Ananworanich J Chomont N Eller LA RV217 and RV254/SEARCH010 study groups. HIV DNA set point is rapidly established in acute HIV infection and dramatically reduced by early ART. EBioMedicine 2016; 11:68–72. 27460436 10.1016/j.ebiom.2016.07.024 PMC5049918

[45] Pinzone MR Graf E Lynch L. Monitoring integration over time supports a role for cytotoxic t lymphocytes and ongoing replication as determinants of reservoir size. J Virol 2016; 90:10436–10445. 27630237 10.1128/JVI.00242-16 PMC5110192

[46] Giron LB Abdel-Mohsen M. Viral and host biomarkers of HIV remission post treatment interruption. Curr HIV/AIDS Rep 2022; 19:217–233. 35438384 10.1007/s11904-022-00607-z

[47] Williams JP Hurst J Stohr W. HIV-1 DNA predicts disease progression and posttreatment virological control. Elife 2014; 3:e03821. 25217531 10.7554/eLife.03821 PMC4199415

[48] Li JZ Etemad B Ahmed H. The size of the expressed HIV reservoir predicts timing of viral rebound after treatment interruption. AIDS 2016; 30:343–353. 26588174 10.1097/QAD.0000000000000953 PMC4840470

[49] Pasternak AO Grijsen ML Wit FW. Cell-associated HIV-1 RNA predicts viral rebound and disease progression after discontinuation of temporary early ART. JCI Insight 2020; 5:134196. 32097124 10.1172/jci.insight.134196 PMC7213790

[50▪] Barbehenn A Shi L Shao J. Rapid biphasic decay of intact and defective HIV DNA reservoir during acute treated HIV disease. Nat Commun 2024; 15:9966. 39557853 10.1038/s41467-024-54116-1 PMC11574060

[51▪] van Paassen PM Pasternak AO Bolluyt DC. HIV-specific CD8+ T-cell proliferative response 24 weeks after early antiretroviral therapy initiation is associated with the subsequent reduction in the viral reservoir. Elife 2026; 14:R106402. 10.7554/eLife.106402 PMC12829989 41575185

[52] Gandhi RT Cyktor JC Bosch RJ AIDS Clinical Trials Group A5321 Team. Selective decay of intact HIV-1 proviral DNA on antiretroviral therapy. J Infect Dis 2021; 223:225–233. 32823274 10.1093/infdis/jiaa532 PMC7857155

[53] Peluso MJ Bacchetti P Ritter KD. Differential decay of intact and defective proviral DNA in HIV-1-infected individuals on suppressive antiretroviral therapy. JCI Insight 2020; 5:132997. 32045386 10.1172/jci.insight.132997 PMC7101154

[54] Singh K Natarajan V Dewar R. Long-term persistence of transcriptionally active ’defective’ HIV-1 proviruses: implications for persistent immune activation during antiretroviral therapy. AIDS 2023; 37:2119–2130. 37555786 10.1097/QAD.0000000000003667 PMC10615727

[55] Imamichi H Dewar RL Adelsberger JW. Defective HIV-1 proviruses produce novel protein-coding RNA species in HIV-infected patients on combination antiretroviral therapy. Proc Natl Acad Sci U S A 2016; 113:8783–8788. 27432972 10.1073/pnas.1609057113 PMC4978246

[56] Imamichi H Smith M Adelsberger JW. Defective HIV-1 proviruses produce viral proteins. Proc Natl Acad Sci U S A 2020; 117:3704–3710. 32029589 10.1073/pnas.1917876117 PMC7035625

[57] Scherpenisse M Kootstra NA Bakker M. Cell-associated HIV-1 unspliced-to-multiply-spliced RNA ratio at 12 weeks of ART predicts immune reconstitution on therapy. mBio 2021; 12:e00099-21. 10.1128/mBio.00099-21 PMC8092199 33688002

[58] Kuniholm J Coote C Henderson AJ. Defective HIV-1 genomes and their potential impact on HIV pathogenesis. Retrovirology 2022; 19:13. 35764966 10.1186/s12977-022-00601-8 PMC9238239

[59] Pasternak AO Tsukamoto T Berkhout B. ’Zombie’ proviruses in the spotlight: exploring the dark side of HIV persistence. AIDS 2023; 37:2239–2241. 37877277 10.1097/QAD.0000000000003721

[60▪] Reddy K Lee GQ Reddy N. Differences in HIV-1 reservoir size, landscape characteristics, and decay dynamics in acute and chronic treated HIV-1 Clade C infection. Elife 2025; 13:R96617. 10.7554/eLife.96617 PMC11841988 39976231

[61▪▪] Pasternak AO van Paassen PM Verschoor YL. Long-term effect of temporary ART initiated during primary HIV-1 infection on viral persistence. Nat Commun 2025; 16:6989. 40738892 10.1038/s41467-025-62362-0 PMC12311192

[62▪] Crowell TA Hsieh HC Wang X Infectious Disease Clinical Research Program HIV Working Group. Antiretroviral therapy within 2 years of HIV acquisition is associated with fewer viral blips: a retrospective analysis of more than 20 years of data from the US Military HIV Natural History Study. Clin Infect Dis 2025; 81:499–509. 40059621 10.1093/cid/ciaf103 PMC12497960

[63] Chun TW Justement JS Murray D. Rebound of plasma viremia following cessation of antiretroviral therapy despite profoundly low levels of HIV reservoir: implications for eradication. AIDS 2010; 24:2803–2808. 20962613 10.1097/QAD.0b013e328340a239 PMC3154092

[64] Trautmann L Mbitikon-Kobo FM Goulet JP. Profound metabolic, functional, and cytolytic differences characterize HIV-specific CD8 T cells in primary and chronic HIV infection. Blood 2012; 120:3466–3477. 22955926 10.1182/blood-2012-04-422550 PMC3743465

[65] Takata H Buranapraditkun S Kessing C. Delayed differentiation of potent effector CD8(+) T cells reducing viremia and reservoir seeding in acute HIV infection. Sci Transl Med 2017; 9:eaag1809. 28202771 10.1126/scitranslmed.aag1809 PMC5678930

[66] Day CL Kaufmann DE Kiepiela P. PD-1 expression on HIV-specific T cells is associated with T-cell exhaustion and disease progression. Nature 2006; 443:350–354. 16921384 10.1038/nature05115

[67] Ndhlovu ZM Kazer SW Nkosi T. Augmentation of HIV-specific T cell function by immediate treatment of hyperacute HIV-1 infection. Sci Transl Med 2019; 11:eaau0528. 31118290 10.1126/scitranslmed.aau0528 PMC6901350

[68] Takata H Kakazu JC Mitchell JL. Long-term antiretroviral therapy initiated in acute HIV infection prevents residual dysfunction of HIV-specific CD8(+) T cells. EBioMedicine 2022; 84:104253. 36088683 10.1016/j.ebiom.2022.104253 PMC9471490

[69] Leong YA Chen Y Ong HS. CXCR5(+) follicular cytotoxic T cells control viral infection in B cell follicles. Nat Immunol 2016; 17:1187–1196. 27487330 10.1038/ni.3543

[70▪] Rueger S Gruener E Wang D. Early treatment and PD1 inhibition enhance HIV-specific functionality of follicular CD8+ T cells. JCI Insight 2025; 10:e180309. 40197363 10.1172/jci.insight.180309 PMC11981630

[71] Pollack RA Jones RB Pertea M. Defective HIV-1 proviruses are expressed and can be recognized by cytotoxic T lymphocytes, which shape the proviral landscape. Cell Host Microbe 2017; 21:494.e4–506.e4. 28407485 10.1016/j.chom.2017.03.008 PMC5433942

[72] Takata H Mitchell JL Pacheco J. An active HIV reservoir during ART is associated with maintenance of HIV-specific CD8(+) T cell magnitude and short-lived differentiation status. Cell Host Microbe 2023; 31:1494–506e4. 37708852 10.1016/j.chom.2023.08.012 PMC10564289

[73▪] Li JZ Melberg M Kittilson A AIDS Clinical Trials Group A5345 Study Team. Predictors of HIV rebound differ by timing of antiretroviral therapy initiation. JCI Insight 2024; 9:e173864. 38329130 10.1172/jci.insight.173864 PMC10967395

[74] Murin CD Wilson IA Ward AB. Antibody responses to viral infections: a structural perspective across three different enveloped viruses. Nat Microbiol 2019; 4:734–747. 30886356 10.1038/s41564-019-0392-y PMC6818971

[75] Richman DD Wrin T Little SJ Petropoulos CJ. Rapid evolution of the neutralizing antibody response to HIV type 1 infection. Proc Natl Acad Sci U S A 2003; 100:4144–4149. 12644702 10.1073/pnas.0630530100 PMC153062

[76] Landais E Moore PL. Development of broadly neutralizing antibodies in HIV-1 infected elite neutralizers. Retrovirology 2018; 15:61. 30185183 10.1186/s12977-018-0443-0 PMC6125991

[77] Wei X Decker JM Wang S. Antibody neutralization and escape by HIV-1. Nature 2003; 422:307–312. 12646921 10.1038/nature01470

[78] Esmaeilzadeh E Etemad B Lavine CL. Autologous neutralizing antibodies increase with early antiretroviral therapy and shape HIV rebound after treatment interruption. Sci Transl Med 2023; 15:eabq4490. 37163616 10.1126/scitranslmed.abq4490 PMC10576978

[79] Levesque MC Moody MA Hwang KK. Polyclonal B cell differentiation and loss of gastrointestinal tract germinal centers in the earliest stages of HIV-1 infection. PLoS Med 2009; 6:e1000107. 19582166 10.1371/journal.pmed.1000107 PMC2702159

[80] Moir S Buckner CM Ho J. B cells in early and chronic HIV infection: evidence for preservation of immune function associated with early initiation of antiretroviral therapy. Blood 2010; 116:5571–5579. 20837780 10.1182/blood-2010-05-285528 PMC3031405

[81▪] Whitehill GD Joy J Marino FE. Autologous neutralizing antibody responses after antiretroviral therapy in acute and early HIV-1. J Clin Invest 2024; 134: 10.1172/JCI176673 PMC11142743 38652564

[82▪▪] Mitchell JL Buranapraditkun S Gantner P. Activation of CXCR3(+) Tfh cells and B cells in lymph nodes during acute HIV-1 infection correlates with HIV-specific antibody development. J Virol 2025; 99:e0153224. 39932316 10.1128/jvi.01532-24 PMC11915809

[83▪] VanderVeen LA Selzer L Moldt B. HIV-1 envelope diversity and sensitivity to broadly neutralizing antibodies across stages of acute HIV-1 infection. AIDS 2024; 38:607–610. 38416554 10.1097/QAD.0000000000003792 PMC10906214

[84] Dong K, Asari V, Govender V, *et al*. Evaluation of 2 bNAbs plus vesatolimod in early-treated South African women with HIV-1 during ATI. Conference on Retroviruses and Opportunistic Infections; San Francisco. 2025.

[85] Lee MJ Cherrill LR Zacharopoulou P. Time to HIV rebound after antiretroviral therapy interruption: a double-blind randomised placebo-controlled trial of long-acting broadly neutralising antibodies; The RIO Trial. medRxiv 2026; 2026.02.04.25342277 [Preprint]. 10.1016/S2352-3018(26)00059-7 42202839

[86▪▪] Peluso MJ Sandel DA Deitchman AN. Correlates of HIV-1 control after combination immunotherapy. Nature 2026; 650:187–195. 41326736 10.1038/s41586-025-09929-5 PMC12872443

[87] Etemad B Sun X Li Y. HIV posttreatment controllers have distinct immunological and virological features. Proc Natl Acad Sci U S A 2023; 120:e2218960120. 36877848 10.1073/pnas.2218960120 PMC10089217

[88▪▪] Kiani Z Urbach JM Wisner H. CD8(+) T cell stemness precedes postintervention control of HIV viremia. Nature 2026; 650:196–204. 41326735 10.1038/s41586-025-09932-w PMC12872466

[89▪] Garcia MA Farrell-Sherman A Zhuo J. Inhibitory potential of autologous neutralizing antibodies sets quantitative limits on the rebound-competent HIV-1 reservoir. bioRxiv 2025; 2025.12.07.692769 [preprint]. 10.1073/pnas.2608337123 PMC13342869 42391404

[90▪] Webb NE Gorman MJ Parker LJ. The functional antibody landscape in HIV posttreatment controllers is heterogeneous. J Virol 2025; 99:e0179025. 41313002 10.1128/jvi.01790-25 PMC12724254

[91] Bartsch YC Loos C Rossignol E. Viral rebound kinetics correlate with distinct HIV antibody features. mBio 2021; 12. 10.1128/mBio.00170-21 PMC8092214 33688003

[92] Molinos-Albert LM Lorin V Monceaux V. Transient viral exposure drives functionally-coordinated humoral immune responses in HIV-1 posttreatment controllers. Nat Commun 2022; 13:1944. 35410989 10.1038/s41467-022-29511-1 PMC9001681

[93] Adams P Berkhout B Pasternak AO. Towards a molecular profile of antiretroviral therapy-free HIV remission. Curr Opin HIV AIDS 2022; 17:301–307. 35938464 10.1097/COH.0000000000000749

